# Supplementary material for: Analysis of PPARγ Signaling Activity in Psoriasis
Source: Int J Mol Sci. 2021 Aug 10;22(16):8603. doi: 10.3390/ijms22168603 (PMC8395241; doi:10.3390/ijms22168603)
Supplement: Supplementary file 1 [file ijms-22-08603-s001.zip › Supplemental materials_Analysis of PPARg signaling activity in psoriasis/Pathway models/Models images and html files/Interleukin-17 and interleukin-22 signaling in psoriasis/9341.html]

MAP2K6


# Protein MAP2K6

|  |  |
| --- | --- |
| URN | urn:agi-llid:5608 |
| Total Entities | 0 |
| Connectivity | 937 |
| Name | MAP2K6 |
| Description | mitogen-activated protein kinase kinase 6 |
| Notes | This gene encodes a member of the dual specificity protein kinase family, which functions as a mitogen-activated protein (MAP) kinase kinase. MAP kinases, also known as extracellular signal-regulated kinases (ERKs), act as an integration point for multiple biochemical signals. This protein phosphorylates and activates p38 MAP kinase in response to inflammatory cytokines or environmental stress. As an essential component of p38 MAP kinase mediated signal transduction pathway, this gene is involved in many cellular processes such as stress induced cell cycle arrest, transcription activation and apoptosis. [provided by RefSeq, Jul 2008] |
| Primary Cell Localization | Cytoplasm |
| Class | Protein kinase |

---

|  |  |
| --- | --- |
| Pathway | Osteoarthritis |
|  | Skin Aging |
|  | Dopamine Receptors Signaling |
|  | Serotonin Receptors Signaling |
|  | Thrombopoietin Receptors Signaling in Platelet Maturation |
|  | Toll-like Receptors Act through MYD88-TIRAP Signaling |
|  | TLR4 Signaling in Leukocytes |
|  | Plasmin Effects in Inflammation |
|  | Platelet Activation via Adhesion Molecules |
|  | Scavenger Receptors in Platelet Activation |
|  | Bradykinin Effects in Inflammation |
|  | MC1R in anti-Inflammatory Signaling |
|  | Leukotriene Effect on Vascular Endothelial Cell Response |
|  | Leukotriene Effect on Airway Smooth Muscle Response |
|  | Basophil Activation |
|  | Mast-Cell Activation without Degranulation through TNFSF8 Signaling |
|  | Mast-Cell Activation via IgE Signaling |
|  | Mast-Cell Activation without Degranulation |
|  | Mast-Cell Activation without Degranulation through IL33/IL1RL1 Signaling |
|  | Mast-Cells De Novo Synthesized Mediators via IgE Independent Signaling |
|  | Mast-Cell Activation without Degranulation through IL1R1 and TLR Signaling |
|  | Macrophage M1 Lineage |
|  | Neutrophil Recruitment and Priming |
|  | Neutrophil Degranulation via FPR1 Signaling |
|  | Vascular Endothelial Cell Activation by Cytokines |
|  | Vascular Endothelial Cell Activation by Growth Factors |
|  | GFs/TNF -> Ion Channels |
|  | Nociception Expression Targets Signaling |
|  | EDNRA/B -> Vascular Motility |
|  | ADRA1A -> IL6 Production |
|  | ADRA1 -> Prostaglandin Generation |
|  | BDKRB1/2 -> Prostaglandin Generation |
|  | CNR1/2 -> IL1B/2/4/6/10 Production |
|  | DRD2 Expression Targets |
|  | CHRM1 -> IL2 Production |
|  | HTR1 -> IL6 Production |
|  | HTR5 -> TNF Production |
|  | HTR1 -> Membrane Transport |
|  | IL1B Expression Targets -> Nociception |
|  | Circadian Clock Genes in Suprachiasmatic Nuclei Neurons |
|  | Cardiomyocyte Dysfunction |
|  | Dendritic Cells Function in Atherosclerosis |
|  | Dilated Cardiomyopathy |
|  | Cardiomyocyte Hypertrophy |
|  | Hypertrophic Cardiomyopathy |
|  | Inflammatory Reaction in Acne Vulgaris |
|  | Keratinocyte Proliferation in Acne Vulgaris |
|  | Mast-Cells Activation in Atopic Dermatitis |
|  | IL17 Signaling in Psoriasis |
|  | Dendritic Cells Function in Psoriasis |
|  | Podocyte Dysfunction in Diabetic Nephropathy |
|  | Mesangial Cell Dysfunction in Diabetic Nephropathy |
|  | Oxidation of Fatty Acid in non-Alcoholic Fatty Liver Disease |
|  | beta-Cell Destruction through Cytokines in Diabetes Mellitus |
|  | Epithelial Cell in the Innate Immune Response in Ulcerative Colitis |
|  | Antiphospholipid Antibodies in Endothelial Cell |
|  | Osteoclast Activation in Multiple Myeloma |
|  | Antiphospholipid Antibodies and Endothelial Cell Interaction |
|  | IL33 Signaling Related Eosinophilia in Asthma |
|  | IgE Induces Airway Smooth Muscle Cell Proliferation |
|  | Basophil Activation in Asthma |
|  | Mast-Cell Activation in Asthma |
|  | Mucin Production in Goblet Airway Epithelial Cells |
|  | Airway Epithelial Cell Dysfunction |
|  | CFTR Expression in Epithelial Cells (Class I Mutations) |
|  | Osteoblast Function Decline in Gout |
|  | Osteoclast Activation in Gout |
|  | TNF and IL1B Induce Metalloproteinase Synthesis in Osteoarthritis |
|  | Apoptosis of Chondrocyte |
|  | IL1B Induced Arthralgia |
|  | Dendritic Cell Dysfunction in Psoriatic Arthritis |
|  | Synovial Fibroblast Activation in Psoriatic Arthritis |
|  | Osteoclast Activation in Rheumatoid Arthritis |
|  | Synovial Fibroblast Activation by Citokines in Rheumatoid Arthritis |
|  | Apoptosis Block in Synovial Fibroblast |
|  | Autocrine Cytokine/Chemokine Loops in Systemic Scleroderma |
|  | Skin Fibrosis |
|  | CCR5 Signaling in Macrophages |
|  | Apoptosis Block in Infected Cells |
|  | Diabetes Induced Periodontitis |
|  | IL1 Receptor Antagonist (IL1RN) Deficiency (DIRA) |
|  | IL36 Receptor Antagonist (IL1F5) Deficiency (DITRA) |
|  | Bone Loss in Osteoporosis |
|  | Hyperparathyroidism, Secondary Effect |
|  | Osteoclast Activation in Postmenopause |
|  | Glioma Invasion Signaling |
|  | Growth Factor Signaling in Neuroblastoma |
|  | Neuroblastoma |
|  | Microglia and Motor Neuron Interaction Dysregulation |
|  | Breast Cancer |
|  | Angiogenesis in Endometriosis |
|  | Subepithelial Fibroblasts in Middle Ear Cholesteatoma |
|  | HPV E5 Regulates EDNRA and EGFR Signaling Pathway in Keratinocytes |
|  | Steroids Induced Cataract |
|  | VEGFA in Neovascular Glaucoma |
|  | Neurotrophic Factor Deprivation in Retinal Ganglion Cell Death |
|  | Cytokines Trigger Otitis Media |
|  | MUC5AC Expression by NTHi and S. Pneumoniae |
|  | Immune System Role in Otitis Media |
|  | Alveolar Macrophages Dysfunction in COPD |
|  | T-Cell Cytotoxic Activation |
|  | Alveolar Epithelial Cell Dysfunction in COPD |
|  | Mucin Hyperproduction in Goblet and Mucous Cells |
|  | Plasmin Role in Glomerulonephritis |
|  | Mesangial Cell Dysfunction in Glomerulonephritis |
|  | Interstitial Fibroblasts in Pyelonephritis |
|  | ANGPT1 -> CREB/CREBBP Expression Targets |
|  | ANGPT2 -> AP-1/CREBBP/MYC Expression Targets |
|  | AREG -> NFATC Expression Target |
|  | AREG -> CREB/CREBBP Expression Targets |
|  | AREG -> AP-1 Expression Targets |
|  | Acetylcholine Expression Targets |
|  | Dopamine/Gi Expression Targets |
|  | BTC -> NFATC Expression Targets |
|  | BTC -> AP-1/ATF/CREB Expression Targets |
|  | CXCR4 -> ARRB1 Signaling |
|  | Sialophorin -> CTNNB/MYC/TP53 Signaling |
|  | CD72 -> NF-kB Expression Targets |
|  | CD8 -> NF-kB Expression Targets |
|  | CD80 -> NFATC Expression Targets |
|  | CD86 -> NFATC Expression Targets |
|  | Complement Component GPCRs Signaling |
|  | Clozapine Induced Granulocytopenia |
|  | EGF -> AP-1/ATF Expression Targets |
|  | EGF -> CREB/CREBBP/ELK/SRF/MYC Expression Targets |
|  | EGF -> MEF/MYOD/NFATC Expression Targets |
|  | EGF -> TP53 Expression Targets |
|  | EGFR/ERBB3 -> MEF/MYOD/NFATC/MYOG Signaling |
|  | EGFR/ERBB2 -> TP53 Signaling |
|  | EGFR -> AP-1/ATF2 Signaling |
|  | EGFR -> AP-1/CREB/ELK/SRF/MYC Signaling |
|  | EREG -> CREB Expression Target |
|  | EREG -> AP-1/ATF Expression Targets |
|  | Erythropoietin -> AP-1/MYC/CREB Expression Targets |
|  | F2 -> AP-1/CREB/ELK/SRF/SP1 Expression Targets |
|  | PLG -> AP-1/CREB/ELK/SRF/SP1 Expression Targets |
|  | FGF9 -> AP-1/CREB/MYC Expression Targets |
|  | FGF10 -> AP-1/CREB/CREBBP/MYC Expression Targets |
|  | FGF1 -> AP-1/CREB/ELK/SRF/MYC Expression Targets |
|  | FGF8 -> AP-1/CREB/MYC Expression Targets |
|  | FGF18 -> AP-1/CREB Expression Targets |
|  | FGF4 -> AP-1/MYC Expression Targets |
|  | FGF7 -> AP-1/CREB/CREBBP/MYC Expression Targets |
|  | FGF2 -> AP-1/CREB/CREBBP/ELK/SRF/MYC Expression Targets |
|  | FGFR -> AP-1/CREB/CREBBP/ELK/SRF/MYC Signaling |
|  | ADCYAP1 Expression Targets |
|  | Glutamate/Gq Expression Targets |
|  | VIP Expression Targets |
|  | HBEGF -> AP-1/ATF Expression Targets |
|  | HBEGF -> TP53 Expression Targets |
|  | HBEGF -> MEF/MYOD Expression Target |
|  | HBEGF -> CREB/MYC Expression Targets |
|  | HGF -> AP-1/CREB/ELK/SRF/MYC Expression Targets |
|  | HGFR -> AP-1/CREB/MYC Signaling |
|  | TGFB Signaling activation by Blocking of Tumor Suppressors |
|  | TGFB Family in Epithelial to Mesenchymal Transition in Cancer |
|  | IGF1 -> MEF/MYOD/MYOG Expression Targets |
|  | IGF2/MEF/MYOD Expression Targets |
|  | Insulin -> MEF/MYOD Expression Targets |
|  | ICAM1 -> AP-1/CREB/ELK/SRF Signaling |
|  | IGF1R -> MEF/MYOD/MYOG Signaling |
|  | FibronectinR -> NF-kB Signaling |
|  | FibronectinR -> AP-1/ELK/SRF/SREBF Signaling |
|  | Fibronectin Expression Targets |
|  | CTGF -> AP-1/CREB/MYC Expression Targets |
|  | ICAM1 Expression Targets |
|  | IL1R -> NF-kB Signaling |
|  | IL16-> ATF/CREB/CREBBP Expression Target |
|  | PAF/Gq -> AP-1/ATF1/CREB/ERK/SRF Expression Targets |
|  | Dronabinol/Anandamide Expression Targets |
|  | PAF/Gq -> NF-kB Expression Targets |
|  | S1P Expression Targets |
|  | NRG1 -> MEF/MYOD Expression Targets |
|  | NRG1 -> CREB/CREBBP/ELK/SRF/MYC Expression Targets |
|  | NRG1 -> TP53 Expression Target |
|  | NRG1 -> AP-1/ATF Expression Targets |
|  | NGF -> SMAD3/NF-kB Expression Targets |
|  | NGF -> AP-1/TP53/MYC Expression Targets |
|  | NGF -> FOXO/MYCN/ELK-SRF Expression Targets |
|  | BDNF Expression Targets |
|  | NTF3 Expression Targets |
|  | NTF4 Expression Targets |
|  | NGF -> CREB/CEBPB/MEF2A Expression Targets |
|  | NOTCH -> TCF3 Signaling |
|  | NOTCH Expression Targets |
|  | PDGFB -> AP-1/CREB/MYC Expression Target |
|  | PDGFC -> CREB Expression Target |
|  | PDGFD -> AP-1 Expression Targets |
|  | PDGF -> AP-1/CREB/CREBBP/MYC Expression Targets |
|  | PDGFR -> AP-1/MYC Signaling |
|  | AVP/Gs -> CREB/ELK/SRF/AP-1/EGR Expression Targets |
|  | EDN1 Expression Targets |
|  | AVP/Gs -> MEF/MYOD/NFATC/MYOG Expression Targets |
|  | AVP/Gq -> MEF/MYOD/NFATC/MYOG Expression Targets |
|  | EDN3 Expression Targets |
|  | AVP/Gq -> CREB/ELK/SRF/AP-1/EGR Expression Targets |
|  | PDCD1 -> ATF/CREB/CREBBP Expression Targets |
|  | AngiopoietinR -> AP-1 Signaling |
|  | NTRK -> AP-1/CREB/ELK/SRF/MYC/SMAD3/TP53 Signaling |
|  | GAS6 -> AP-1/CREB Expression Targets |
|  | FIGF -> AP-1 Expression Target |
|  | KITLG -> AP-1/CREB/CREBBP/MYC Expression Targets |
|  | CSF1 -> AP-1/CREB/CREBBP/MYC Expression Targets |
|  | PGF -> AP-1/CREB/CREBBP/MYC Expression Targets |
|  | FLT3LG -> AP-1/CREB/CREBBP Expression Targets |
|  | TGFBR -> AP-1 Signaling |
|  | TGFBR -> CREB/ELK-SRF Signaling |
|  | TGFBR -> ATF/GADD/MAX/TP53 Signaling |
|  | TGFBR -> MEF/MYOD/MYOG Signaling |
|  | TNF-alpha/TNFRSF1A Signaling |
|  | TGF-beta Signaling |
|  | P38 MAPK/MAPK14 Signaling |
|  | VEGF Signaling |
|  | TCR -> CREB/CREBBP/ATF Expression Targets |
|  | T-Cell Receptor -> ATF/CREB Signaling |
|  | TGFA -> TP53 Expression Targets |
|  | TGFA -> MEF/MYOD/NFATC Expression Targets |
|  | TGFA/AP-1/ATF Expression Targets |
|  | TGFA -> CREB/CREBBP/ELK-SRF/MYC Expression Targets |
|  | TGFB3-TGFBR2 Expression Targets |
|  | TGFB2-TGFBR2 Expression Targets |
|  | TGFB1-TGFBR2 Expression Targets |
|  | FASLG Expression Targets |
|  | TNF -> NF-kB Expression Targets |
|  | TNFSF10 Expression Targets |
|  | LTA Expression Targets |
|  | TNFSF14 Expression Targets |
|  | TNF -> AP-1 Expression Targets |
|  | CD72 -> AP-1 Expression Targets |
|  | CD40LG -> NF-kB/ELK/SRF -> CREB/NFATC Expression Targets |
|  | TNFSF13 Expression Targets |
|  | TNFSF13B Expression Targets |
|  | TNF -> ELK-SRF Expression Targets |
|  | TNF -> CREB Expression Targets |
|  | TNF -> TP53/ATF Expression Targets |
|  | EDA Expression Targets |
|  | EctodysplasinR -> NF-kB Signaling |
|  | TNFR -> CREB/ELK-SRF Signaling |
|  | TNFR -> AP-1/ATF/TP53 Signaling |
|  | NGFR -> AP-1/CEBPB/CREB/ELK/SRF/TP53 Signaling |
|  | TNFRSF5/6 -> RB1/E2F Signaling |
|  | NGFR -> NF-kB Signaling |
|  | TNFR -> NF-kB Signaling |
|  | NGFR -> MEF Signaling |
|  | TNFRSF6 -> DDIT3 Signaling |
|  | TNFRSF1A -> AP-1/ATF/TP53 Signaling |
|  | TNFRSF1A -> CREB/ELK-SRF Signaling |
|  | Thrombopoietin -> AP-1/CREB/CREBBP/MYC Expression Targets |
|  | TLR4 -> AP-1 Expression Targets |
|  | TLR5 Expression Targets |
|  | TLR7 Expression Targets |
|  | TLR4 -> AP-1/EGR1/HIF1A Expression Targets |
|  | TLR1 -> 2/6 Expression Targets |
|  | IL1B Expression Targets |
|  | IL1A Expression Targets |
|  | TLR9 Expression Targets |
|  | TLR -> AP-1 Signaling |
|  | ER Stress (Unfolded Protein Response) |
|  | ROS in Angiotensin Mediated Cardiovascular Remodeling and Hypertrophy |
|  | VEGFA -> ATF/CREB/ELK-SRF Expression Targets |
|  | VEGFA -> NFATC Expression Targets |
|  | VEGFC -> ATF Expression Target |
|  | VEGFA -> AP-1/CREBBP/MYC Expression Targets |
|  | VEGFR -> NFATC Signaling |
|  | VEGFR -> AP-1/CREB/MYC Signaling |
|  | VEGFR -> ATF/CREB/ELK-SRF Signaling |
|  | 1\_Inflammation in keratinocytes in the pathology of acne |
|  | 2\_Interleukin-17 and interleukin-22 signaling in psoriasis |
|  | 1\_Osteoclast-mediated bone resorption in postmenopausal women |
|  | 3\_The role of interstitial fibroblasts in pyelonephritis |
|  | 3\_Mesangial cell dysfunction in glomerulonephritis |
|  | 3\_Role of neurotrophic factor deprivation in retinal ganglion cell death |
|  | 2\_Pathogens stimulate mucins expression in the middle ear |
|  | 3\_Arterial wall inflammation and calcification in atherosclerosis |
|  | 2\_Cardiomyocyte hypertrophy |
|  | 3\_Cardiomyocyte dysfunction unrelated to cell junctions in ARVD |
|  | 1\_Cigarette smoke causes dysfunction and production of inflammatory mediators in alveolar epithelial cells |
|  | 3\_Role of alveolar macrophages in pulmonary tissue destruction in COPD |
|  | 4\_2\_Insulin resistance: inflammation-related insulin resistance |
|  | mapk1 protein modificators |
|  | Interleukin-17 and interleukin-22 signaling in psoriasis |

---

|  |  |
| --- | --- |
| Source | curated |

---

|  |  |
| --- | --- |
| MedScan ID | 5608 |

---

|  |  |
| --- | --- |
| LocusLink ID | 5608 |
|  | 26399 |
|  | 114495 |
|  | 19104 |
|  | 286883 |
|  | 568951 |

---

|  |  |
| --- | --- |
| Alias | MEK6 |
|  | ERK kinase VI |
|  | Dual specificity mitogen-activated protein kinase kinase 6 |
|  | MAPK/ERK kinase VI |
|  | Map2k6 |
|  | SAPK kinase 3 |
|  | MAPKK6 |
|  | protein kinase, mitogen-activated, kinase VI |
|  | PRKMK6 |
|  | MGC93287 |
|  | OTTMUSP00000003317 |
|  | Dual specificity mitogen-activated protein kinase kinase VI |
|  | RP23-162E10.1 |
|  | MAP kinase kinase 6 |
|  | MAPK kinase 6 |
|  | OTTMUSP00000003318 |
|  | MAPK kinase VI |
|  | MAP kinase kinase VI |
|  | MAPK/ERK kinase 6 |
|  | protein kinase, mitogen activated, kinase 6 |
|  | MKK6 |
|  | ERK kinase 6 |
|  | stress activated protein kinase kinase 3 |
|  | SAPKK3 |
|  | mitogen-activated protein kinase kinase VI |
|  | mitogen activated protein kinase kinase 6 |
|  | protein kinase, mitogen-activated, kinase 6 (MAP kinase kinase 6) |
|  | SAPKK-3 |
|  | MAPKK 6 |
|  | MEK 6 |
|  | stress-activated protein kinase kinase 3 |
|  | SKK3 |
|  | mitogen-activated protein kinase kinase 6 |
|  | protein kinase, mitogen-activated, kinase 6 |
|  | PRKMK6s |
|  | MAPKK6s |
|  | SAPKK3s |
|  | kinase 6 |

---

|  |  |
| --- | --- |
| EC Number | 2.7.12.2 |
|  | 2.7.1.- |

---

|  |  |
| --- | --- |
| GO ID | 0005524 |
|  | 0004708 |
|  | 0019901 |
|  | 0004674 |
|  | 0004713 |
|  | 0006975 |
|  | 0000165 |
|  | 0000187 |
|  | 0032147 |
|  | 0006915 |
|  | 0060048 |
|  | 0007050 |
|  | 0072709 |
|  | 0120163 |
|  | 0070423 |
|  | 0022602 |
|  | 0018108 |
|  | 0043065 |
|  | 0051770 |
|  | 0032308 |
|  | 0042493 |
|  | 0002931 |
|  | 0007165 |
|  | 0023014 |
|  | 0031098 |
|  | 0005737 |
|  | 0005856 |
|  | 0005829 |
|  | 0005654 |
|  | 0005634 |
|  | 0016301 |
|  | 0000166 |
|  | 0004672 |
|  | 0016740 |
|  | 0016310 |
|  | 0001934 |
|  | 0006468 |
|  | 0004702 |
|  | 0035897 |
|  | 0006355 |
|  | 0006351 |
|  | 0006950 |
|  | 0007346 |
|  | 0002755 |
|  | 0002756 |
|  | 0035666 |
|  | 0045087 |
|  | 0042692 |
|  | 0035872 |
|  | 0009405 |
|  | 0051149 |
|  | 0051403 |
|  | 0034166 |
|  | 0034134 |
|  | 0034138 |
|  | 0034142 |
|  | 0034146 |
|  | 0034162 |
|  | 0038123 |
|  | 0038124 |
|  | 0002224 |
|  | 0016772 |
|  | 0005622 |
|  | 0035556 |
|  | 0008063 |
|  | 0034130 |
|  | 0008152 |
|  | 0005515 |

---

|  |  |
| --- | --- |
| KEGG ID | hsa:5608 |
|  | mmu:26399 |
|  | rno:114495 |

---

|  |  |
| --- | --- |
| MedLine Reference | 10848581 |
|  | 8621675 |
|  | 12213961 |
|  | 12556533 |
|  | 14744933 |
|  | 11593045 |
|  | 12477932 |
|  | 12829175 |
|  | 14709549 |
|  | 15492008 |
|  | 15722372 |
|  | 16755153 |
|  | 17007737 |
|  | 10097111 |
|  | 10349636 |
|  | 10922068 |
|  | 10949032 |
|  | 11042159 |
|  | 11076861 |
|  | 11217851 |
|  | 12151339 |
|  | 12466851 |
|  | 12482988 |
|  | 12824301 |
|  | 12893778 |
|  | 15644321 |
|  | 15767678 |
|  | 16141072 |
|  | 16141073 |
|  | 16199873 |
|  | 16387856 |
|  | 16498455 |
|  | 8861944 |
|  | 10497253 |
|  | 10570156 |
|  | 10961885 |
|  | 11062067 |
|  | 11279118 |
|  | 11460167 |
|  | 11727828 |
|  | 11836244 |
|  | 11971971 |
|  | 12054652 |
|  | 12509443 |
|  | 12589052 |
|  | 12761180 |
|  | 15375157 |
|  | 15516490 |
|  | 15550393 |
|  | 15677464 |
|  | 15778394 |
|  | 15790570 |
|  | 15866172 |
|  | 16313339 |
|  | 16342939 |
|  | 16344560 |
|  | 16728640 |
|  | 7750576 |
|  | 8622669 |
|  | 8626699 |
|  | 8663074 |
|  | 9218798 |
|  | 9373149 |
|  | 9430721 |
|  | 9768359 |
|  | 9841871 |
|  | 11282978 |
|  | 16305752 |
|  | 11104681 |
|  | 15489334 |
|  | 16960152 |
|  | 8125298 |
|  | 17214902 |
|  | 17588539 |

---

|  |  |
| --- | --- |
| Organism | Homo sapiens {Organism urn:agi-taxid:9606} |
|  | Mus musculus {Organism urn:agi-taxid:10090} |
|  | Rattus norvegicus {Organism urn:agi-taxid:10116} |
|  | Homo sapiens |
|  | Mus musculus |
|  | Rattus norvegicus |
|  | Bos taurus |
|  | Danio rerio |

---

|  |  |
| --- | --- |
| Mouse chromosome position | 11 |
|  | 11 E1 |

---

|  |  |
| --- | --- |
| OMIM ID | 601254 |
|  | OMIM:601254 |

---

|  |  |
| --- | --- |
| Rat chromosome position | 10q32.1 |

---

|  |  |
| --- | --- |
| Hugo ID | 6846 |
|  | HGNC:6846 |

---

|  |  |
| --- | --- |
| Human chromosome position | 17q24.3 |

---

|  |  |
| --- | --- |
| Swiss-Prot Accession | A0A024R8K3 |
|  | A8K3Y2 |
|  | P52564 |
|  | P52564.1 |
|  | P70236 |
|  | Q543Z5 |
|  | P70236.1 |
|  | Q925D6 |
|  | Q60521 |
|  | Q6MZH7 |
|  | P70273 |
|  | Q3UU57 |
|  | Q8BP44 |
|  | Q8C371 |
|  | MP2K6\_HUMAN |
|  | MP2K6\_MOUSE |
|  | A8K3Y2\_HUMAN |
|  | Q543Z5\_MOUSE |
|  | Q925D6\_RAT |

---

|  |  |
| --- | --- |
| PIR ID | S71631 |
|  | S71632 |

---

|  |  |
| --- | --- |
| GenBank ID | NC\_000017 |
|  | NM\_002758 |
|  | NP\_002749 |
|  | XM\_005257516 |
|  | XP\_005257573 |
|  | XM\_011525027 |
|  | XP\_011523329 |
|  | NM\_001330450 |
|  | NP\_001317379 |
|  | XM\_011525026 |
|  | XP\_011523328 |
|  | XM\_006721975 |
|  | XP\_006722038 |
|  | NG\_029437 |
|  | AC002546 |
|  | AC015920 |
|  | CH471099 |
|  | EAW89084 |
|  | EAW89085 |
|  | EAW89086 |
|  | EU332850 |
|  | ABY87539 |
|  | JX512448 |
|  | AGC09595 |
|  | KT585201 |
|  | AA932919 |
|  | AB451252 |
|  | BAG70066 |
|  | AB451376 |
|  | BAG70190 |
|  | AK056975 |
|  | AK225719 |
|  | AK290747 |
|  | BAF83436 |
|  | BC012009 |
|  | AAH12009 |
|  | BC037328 |
|  | BX641121 |
|  | CAE46056 |
|  | D87905 |
|  | BAA13496 |
|  | DB111443 |
|  | U39064 |
|  | AAB03708 |
|  | U39065 |
|  | AAB03705 |
|  | U39656 |
|  | AAC50388 |
|  | U39657 |
|  | AAC50389 |
|  | U49732 |
|  | AAB05035 |
|  | X96757 |
|  | CAA65532 |
|  | P52564 |
|  | NC\_000077 |
|  | XM\_011249034 |
|  | XP\_011247336 |
|  | NM\_011943 |
|  | NP\_036073 |
|  | XM\_011249035 |
|  | XP\_011247337 |
|  | XM\_006533378 |
|  | XP\_006533441 |
|  | XM\_006533377 |
|  | XP\_006533440 |
|  | NM\_001356345 |
|  | NM\_001356346 |
|  | NM\_001356347 |
|  | NM\_001356352 |
|  | NM\_001356353 |
|  | AL691461 |
|  | CH466558 |
|  | EDL34384 |
|  | EDL34385 |
|  | EDL34386 |
|  | EDL34387 |
|  | AA389344 |
|  | AI429449 |
|  | AK043482 |
|  | BAC31556 |
|  | AK053547 |
|  | AK077712 |
|  | BAC36972 |
|  | AK086722 |
|  | BAC39729 |
|  | AK138757 |
|  | BAE23771 |
|  | AK140289 |
|  | AK141708 |
|  | BAE24808 |
|  | AK162671 |
|  | BAE37014 |
|  | BB202796 |
|  | BE859531 |
|  | BU695367 |
|  | BY280286 |
|  | BY284275 |
|  | U39066 |
|  | AAB03709 |
|  | X97052 |
|  | CAA65764 |
|  | X98067 |
|  | CAA66670 |
|  | P70236 |
|  | NC\_005109 |
|  | NM\_053703 |
|  | NP\_446155 |
|  | AC\_000078 |
|  | AABR07030670 |
|  | AABR07030671 |
|  | AABR07030672 |
|  | AABR07030673 |
|  | AAHX01066462 |
|  | AAHX01066463 |
|  | AAHX01066464 |
|  | AAHX01066465 |
|  | AAHX01066466 |
|  | AAHX01066467 |
|  | CH473948 |
|  | EDM06489 |
|  | EDM06490 |
|  | EDM06491 |
|  | AF369384 |
|  | AAK53428 |
|  | BC087004 |
|  | AAH87004 |
|  | XM\_005257515 |
|  | XP\_005257572 |
|  | XM\_011525025 |
|  | XP\_011523327 |
|  | NC\_018928 |
|  | AMYH02033884 |
|  | AMYH02033885 |
|  | DQ891488 |
|  | ABM82414 |
|  | DQ894673 |
|  | ABM85599 |
|  | XM\_011249036 |
|  | XP\_011247338 |
|  | XM\_006533376 |
|  | XP\_006533439 |
|  | AC\_000033 |
|  | AAHY01096284 |
|  | AAHY01096285 |
|  | AAHY01096286 |
|  | AAHY01096287 |
|  | AAHY01096288 |
|  | AAHY01096289 |
|  | BC075652 |
|  | AAH75652 |
|  | XM\_005257517 |
|  | XP\_005257574 |
|  | AC\_000149 |
|  | ABBA01028938 |
|  | ABBA01028939 |
|  | ABBA01028940 |
|  | ABBA01028941 |
|  | ABBA01028942 |
|  | ABBA01028943 |
|  | ABBA01028944 |
|  | XM\_006533375 |
|  | XP\_006533438 |
|  | AABR06065948 |
|  | AABR06065949 |
|  | AABR06065950 |
|  | AABR06065951 |
|  | AABR06065952 |
|  | AABR06065953 |
|  | AABR06065954 |
|  | AABR06065955 |
|  | AL603826 |
|  | CAM17458 |
|  | CAM15928 |
|  | CAM15929 |
|  | NT\_010783 |
|  | NW\_001838454 |
|  | NW\_926918 |
|  | AC\_000060 |
|  | CR604345 |
|  | CR616398 |
|  | CR624804 |
|  | CR625265 |
|  | Q6MZH7 |
|  | NT\_165773 |
|  | NW\_001030434 |
|  | P70273 |
|  | Q3UU57 |
|  | Q543Z5 |
|  | Q60521 |
|  | Q8BP44 |
|  | Q8C371 |
|  | NW\_047343 |
|  | NW\_001084656 |
|  | Q925D6 |
|  | NT\_010641 |
|  | NM\_031988 |
|  | NT\_096135 |
|  | BG065128 |
|  | NP\_114365 |
|  | NW\_000040 |
|  | BB261602 |
|  | BG078419 |
|  | 34875497 |
|  | NT\_086886 |
|  | NT\_039521 |
|  | 56799416 |
|  | 16758520 |
|  | 14039929 |
|  | 14039930 |
|  | 56270320 |
|  | 56270321 |
|  | 51474120 |
|  | 14589899 |
|  | 14589900 |
|  | 14589901 |
|  | 14589902 |
|  | 51474508 |
|  | 15080539 |
|  | 15080540 |
|  | 1596164 |
|  | 1596165 |
|  | 1209670 |
|  | 1209671 |
|  | 1209672 |
|  | 1408567 |
|  | 1203815 |
|  | 1203816 |
|  | 1203817 |
|  | 1203818 |
|  | 1314476 |
|  | 1314477 |
|  | 1495484 |
|  | 1495485 |
|  | 1709088 |
|  | 51766637 |
|  | 33859653 |
|  | 33859654 |
|  | 26335710 |
|  | 26335711 |
|  | 26346646 |
|  | 26346647 |
|  | 49523345 |
|  | 49523346 |
|  | 1209674 |
|  | 1209675 |
|  | 1495701 |
|  | 1495702 |
|  | 1495709 |
|  | 1495710 |
|  | 3024165 |
|  | 81916439 |
|  | 123788527 |
|  | 26352185 |
|  | 74200485 |
|  | 74202136 |
|  | 74218281 |
|  | 81883904 |
|  | 81908495 |
|  | 81909188 |
|  | 81913452 |
|  | 81913971 |
|  | 119609491 |
|  | 123995995 |
|  | 1667613 |
|  | 59857961 |
|  | 75039372 |
|  | 75070059 |
|  | AAB18824 |
|  | AAX08815 |
|  | BT020798 |
|  | NC\_007317 |
|  | NM\_001034045 |
|  | NP\_001029217 |
|  | NW\_001493708 |
|  | P79118 |
|  | Q5E9X2 |
|  | U76873 |
|  | 144686011 |
|  | ABP01909 |
|  | EF489398 |
|  | NC\_007123 |
|  | NW\_001510704 |
|  | XM\_692311 |
|  | XP\_697403 |
|  | 119609490 |
|  | 119609492 |
|  | 123981170 |
|  | 34365448 |
|  | 123227427 |
|  | 148702437 |
|  | 148702438 |
|  | 148702439 |
|  | 148702440 |
|  | 149054672 |
|  | 149054673 |
|  | 149054674 |

---

|  |  |
| --- | --- |
| Swiss-Prot ID | MP2K6\_HUMAN |
|  | MP2K6\_MOUSE |
|  | A8K3Y2\_HUMAN |
|  | Q543Z5\_MOUSE |
|  | Q925D6\_RAT |

---

|  |  |
| --- | --- |
| Cell Localization | Nucleus |
|  | Cytoplasm |
|  | Cytoskeleton |
|  | Cytoplasm, cytoskeleton |

---

|  |  |
| --- | --- |
| Ensembl ID | ENSG00000108984 |
|  | ENSP00000467213.1 |
|  | ENST00000589647.5 |
|  | ENSP00000468348.1 |
|  | ENST00000590474.7 |
|  | ENSMUSG00000020623 |
|  | ENSMUSP00000020949.5 |
|  | ENSMUST00000020949.11 |
|  | ENSRNOG00000004437 |
|  | ENSRNOP00000006217.3 |
|  | ENSRNOT00000006217.6 |
|  | ENSP00000468348 |
|  | ENST00000590474 |
|  | ENSP00000467213 |
|  | ENST00000589647 |
|  | ENSMUSP00000020949 |
|  | ENSMUST00000020949 |
|  | ENSRNOP00000006217 |
|  | ENSRNOT00000006217 |

---

|  |  |
| --- | --- |
| MGI ID | MGI:1346870 |
|  | 1346870 |
|  | 108032 |

---

|  |  |
| --- | --- |
| RGD ID | 620666 |

---

|  |  |
| --- | --- |
| Unigene ID | Hs.463978 |
|  | Mm.14487 |
|  | Rn.17256 |
|  | Hs.256924 |
|  | Bt.561 |
|  | Dr.88531 |

---

|  |  |
| --- | --- |
| Homologene ID | 55686 |

---

|  |  |
| --- | --- |
| Shape | Sickle-vertex |

---

|  |  |
| --- | --- |
| IPI ID | IPI00003814 |
|  | IPI00219569 |
|  | IPI00137439 |
|  | IPI00205537 |
|  | IPI00425919 |
|  | IPI00796408 |
|  | IPI00648735 |
|  | IPI00781935 |

---

|  |  |
| --- | --- |
| Microarray ID | 849 |
|  | OH7912 |
|  | MB172 |
|  | 1387809\_at |
|  | rc\_ai176689\_at |
|  | A\_42\_P748910 |
|  | A\_51\_P256093 |
|  | A\_52\_P299915 |
|  | U39064 |
|  | 76095\_at |
|  | 205699\_at |
|  | 205698\_s\_at |
|  | 1543\_at |
|  | 2014\_s\_at |
|  | U39657\_at |
|  | A\_23\_P207445 |
|  | A\_14\_P106514 |
|  | A\_14\_P132828 |
|  | A\_14\_P133360 |
|  | A\_24\_P416489 |
|  | A\_14\_P106339 |
|  | A\_14\_P130621 |
|  | M80 |
|  | 102828\_at |
|  | 102829\_s\_at |

---

|  |  |
| --- | --- |
| Homo sapiens Chromosome position | 17q24.3 |

---

|  |  |
| --- | --- |
| Ensembl Transcript ID | ENST00000359094 |

---

|  |  |
| --- | --- |
| FunctionalClass | MAP kinase kinase |
|  | protein binding |
|  | ATP binding |
|  | kinase |
|  | nucleotide binding |
|  | protein kinase |
|  | protein serine/threonine kinase |
|  | protein-tyrosine kinase |
|  | transferase |

---

|  |  |
| --- | --- |
| Hugo Symbol | MAP2K6 |

---

|  |  |
| --- | --- |
| Bos taurus Chromosome position | 19 |

---

|  |  |
| --- | --- |
| Mus musculus Chromosome position | 11 E1 |

---

|  |  |
| --- | --- |
| KEGG pathway | Toll-like receptor signaling pathway |
|  | MAPK signaling pathway |
|  | Fc epsilon RI signaling pathway |
|  | GnRH signaling pathway |

---

|  |  |
| --- | --- |
| Rattus norvegicus Chromosome position | 10q32.1 |

---

|  |  |
| --- | --- |
| Ensembl Gene ID | ENSG00000108984 |

---

|  |  |
| --- | --- |
| ProteinAtlas NormalTissue | Tissue: cerebral cortex; Cell type: glial cells; Level: Medium; Expression type: APE; Reliability: Medium |
|  | Tissue: lymph node; Cell type: non-germinal center cells; Level: Medium; Expression type: APE; Reliability: Medium |
|  | Tissue: cerebral cortex; Cell type: neuronal cells; Level: Low; Expression type: APE; Reliability: Medium |

---
